# Chlorine redox chemistry is widespread in microbiology

**DOI:** 10.1038/s41396-022-01317-5

**Published:** 2022-10-06

**Authors:** Tyler P. Barnum, John D. Coates

**Affiliations:** Department of Plant and Microbial Biology, University of California, Berkeley, CA 94720 USA

**Keywords:** Soil microbiology, Water microbiology

## Abstract

Chlorine is abundant in cells and biomolecules, yet the biology of chlorine oxidation and reduction is poorly understood. Some bacteria encode the enzyme chlorite dismutase (Cld), which detoxifies chlorite (ClO_2_^−^) by converting it to chloride (Cl^−^) and molecular oxygen (O_2_). Cld is highly specific for chlorite and aside from low hydrogen peroxide activity has no known alternative substrate. Here, we reasoned that because chlorite is an intermediate oxidation state of chlorine, Cld can be used as a biomarker for oxidized chlorine species. Cld was abundant in metagenomes from various terrestrial habitats. About 5% of bacterial and archaeal genera contain a microorganism encoding Cld in its genome, and within some genera Cld is highly conserved. Cld has been subjected to extensive horizontal gene transfer. Genes found to have a genetic association with Cld include known genes for responding to reactive chlorine species and uncharacterized genes for transporters, regulatory elements, and putative oxidoreductases that present targets for future research. Cld was repeatedly co-located in genomes with genes for enzymes that can inadvertently reduce perchlorate (ClO_4_^−^) or chlorate (ClO_3_^−^), indicating that in situ (per)chlorate reduction does not only occur through specialized anaerobic respiratory metabolisms. The presence of Cld in genomes of obligate aerobes without such enzymes suggested that chlorite, like hypochlorous acid (HOCl), might be formed by oxidative processes within natural habitats. In summary, the comparative genomics of Cld has provided an atlas for a deeper understanding of chlorine oxidation and reduction reactions that are an underrecognized feature of biology.

## Introduction

Chlorine is converted between organic and inorganic forms in a biogeochemical cycle [1]. Oxidation and reduction of chlorine produces different inorganic species, including the chlorine oxyanions hypochlorite (ClO^−^) (and its conjugate acid hypochlorous acid, HOCl), chlorite (ClO_2_^−^), chlorate (ClO_3_^−^), and perchlorate (ClO_4_^−^) [2–7]. In biology, oxidized chlorine is presently understood to be a source of energy (ClO_4_^−^, ClO_3_^−^), an intermediate in chlorination (HOCl), a chemical weapon (HOCl), and a source of oxidative stress (ClO_3_^−^, ClO_2_^−^, HOCl) [2, 6, 8, 9]. This biology reflects the high reduction potential of oxidized chlorine and the higher reactivity of its lower oxidation states: perchlorate is stable in aqueous solution, but hypochlorous acid is very reactive. Compared to other elements, less is known about the processes that produce and consume oxidized chlorine and how oxidized chlorine interacts with biology.

The production of oxidized chlorine species within biological habitats depends on oxidation state. Hypochlorous acid can be produced intracellularly and extracellularly from chemical or biochemical oxidation of chloride by enzymes like chloroperoxidase [4, 5, 9–11]. However, no biological oxidation of chlorine to chlorite has been observed, likely due to the high reduction potential of the redox half-reactions involved (*E*^*0*^*’* > 1 V) [7]. While (photo)chemical oxidation of aqueous hypochlorous acid to chlorate and perchlorate has been observed experimentally [12], production of perchlorate and chlorate is thought to occur predominantly in the atmosphere [7, 13, 14]. The degree of oxidation that occurs within biological habitats could be clarified by identifying the chlorine species encountered by microorganisms from different habitats.

The consumption of oxidized chlorine species, aside from the highly reactive hypochlorous acid, is thought to occur predominantly through dissimilatory (per)chlorate reduction, a specialized anaerobic respiratory pathway wherein high-affinity perchlorate reductases (Pcr) or chlorate reductases (Clr) reduce perchlorate or chlorate to provide energy in anoxic habitats [7, 15, 16]. Reduction may instead occur through co-metabolism: due to the structural and chemical similarity between oxyanions like nitrate and chlorate and perchlorate, enzymes such as nitrate reductase can reduce perchlorate or chlorate [17–21]. Laboratory studies have shown this inadvertent reduction of perchlorate or chlorate produces chlorite and damages cells unless chlorite is degraded [22]. An unanswered question is if co-metabolic (per)chlorate reduction occurs at a meaningful extent at the low concentrations of perchlorate and chlorate found in natural environments. If so, many more organisms would contribute to perchlorate and chlorate reduction than presently understood.

A promising approach to answer these questions is to use a biomarker for oxidized chlorine molecules. Chlorite dismutase (Cld) is a heme-containing enzyme that catalyzes a chlorite: oxygen lyase reaction wherein a single molecule of chlorite is cleaved into chloride and molecular oxygen, which detoxifies chlorite and yields oxygen [23–25]. First identified as necessary enzyme in canonical dissimilatory (per)chlorate-reducing bacteria [26, 27], Cld has since been found in bacteria not known to produce chlorite as part of their metabolism [28]. Subsequent investigations have defined the amino acids required for Cld activity [29] and found that aside from low hydrogen peroxidase activity, Cld has no activity towards other compounds, including nitrite, nitric oxide, hydroxylamine, and thiocyanate [28, 30]. These properties make the gene *cld* a useful, specific biomarker for chlorite. Because chlorite is an intermediate oxidation state of chlorine, microorganisms encoding Cld in their genomes have likely experienced not only chlorite but also more-oxidized chlorine species that can be reduced to chlorite and more-reduced chlorine oxyanion species to which chlorite is reduced.

Here, we use *cld* as a biomarker for chlorite in microbial genomes to expand what is known about the biology of chlorine oxyanions and redox chemistry. This comparative genomics approach adopts only two assumptions: that microorganisms encoding Cld experienced chlorite, and that genetic proximity to *cld* means a gene is more likely to be functionally related to *cld* [31, 32]. Beyond expanding the biology of chlorine oxidation and reduction, these results provide an extensive catalogue of genes potentially involved in chlorine biology for future research.

## Results and Discussion

### Distribution of Cld

**Fig. 1:**
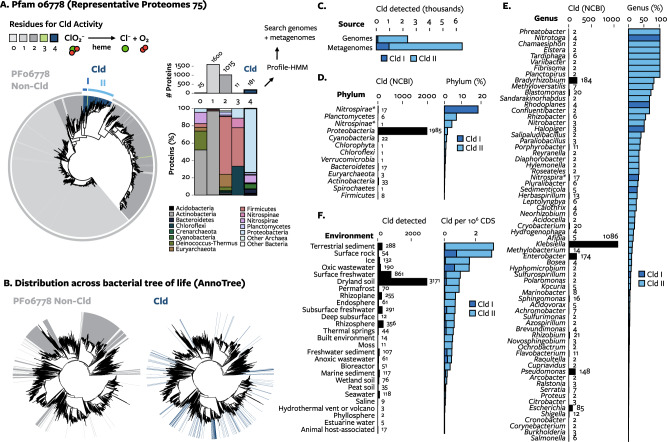
The distribution of Cld across genomes and metagenomes. **A** A maximum-likelihood phylogenetic tree of Pfam 06778, rooted to match Zámocký, Hofbauer [34]. Color indicates the number of the 4 key residues for Cld activity in each protein. The number of proteins with each fraction of key residues, and the phylogenetic distribution of those proteins, is summarized at right. **B** A tree of all bacterial genomes annotated with the presence Pfam 06778 proteins, comparing the distribution of non-Cld proteins (left, gray) and Cld proteins (right, blue). Other domains are not shown because Cld is sparsely distributed. **C** The total number of each Cld lineage detected in genomes and metagenomes. **D**, **E** The number (left) and percent (right) genomes within a given RefSeq phylum or genus. For simplicity, only genera with more than one genome encoding Cld and either 20+ genomes or >20% genomes encoding Cld are shown. Groups with low number and high percent of genomes with Cld likely have an overestimated percentage. The frequency of Cld in *Nitrospirae* and *Nitrospinae* may be underestimated due to the large number of incomplete metagenome-derived genomes in these phyla; these taxa are denoted by asterisks. **F** The number of Cld (left) and fraction of *cld* per million genes (right) in different environments. Only environments with a sample size of more than 10 million genes are shown. Assuming an average of 5000 genes per bacterial genome, 1 cld per 1,000,000 genes means that roughly 0.5% of bacterial genomes in a habitat encode Cld.

Cld proteins belong to the protein family Pfam 06778 [33]. Non-Cld proteins in Pfam 06778, from which Cld evolved [34], are mostly iron-coproporphyrin oxidative decarboxylases (HemQ) required for heme biosynthesis in monoderm bacteria [35]. The use of chlorite dismutase (Cld) as a biomarker requires an accurate definition of proteins with Cld activity, as non-Cld proteins are often incorrectly annotated as Cld or Cld-like proteins in public databases. Here, Cld was defined as proteins in Pfam 06778 that contain the key residues required for Cld activity [23, 29]. Cld proteins formed a monophyletic clade (Fig. 1A), consisting of two major lineages, confirming previous analyses with smaller datasets [22, 34, 36, 37]. Cld proteins were primarily found in diderm phyla (Fig. 1A) and were sparsely distributed across the tree of life (Fig. 1B).

An expansive search for Cld identified 2411 Cld proteins in 2297 genomes/metagenome-assembled genomes and 6469 Cld in 1575 metagenomes (Fig. 1C, Supplementary Data). Cld was identified in 14 phyla and 143 genera, including the bacterial phyla *Actinobacteria*, *Verrucomicrobia*, *Firmicutes*, *Chloroflexi*, and *Spirochaetes* in which Cld has not previously been reported (Supplementary Data). For the first time, Cld was identified in the *Archaea* and *Eukarya*. The low percent identity to bacterial Cld sequences and the similarity of neighboring genes to non-bacterial genes corroborated their assignment to these taxa. The eukaryote with Cld was the unicellular green alga *Monoraphidium neglectum* [38]. Cld was previously reported in a different eukaryote, the poplar tree (*Populus*) [37], but this was later determined to be contamination by bacterial genomic DNA and removed (personal communication, Joint Genome Institute).

Overall, Cld was observed in approximately 1% of genomes, 5% of genera and 15% of phyla in the NCBI taxonomy among the prokaryotes sampled. Genomes from the phyla *Nitrospirae, Planctomycetes*, and *Nitrospinae* are most likely to contain Cld, followed by *Proteobacteria* and *Cyanobacteria* (Fig. 1D). At the genus level, typically only a fraction of genomes had Cld, although Cld could be highly conserved within a genus (Fig. 1E). The widespread nature of Cld was further supported by its distribution across a dataset of 6961 IMG/M metagenomes encoding 10.8 billion genes. *cld* were a very low proportion of genes in host-associated systems and a greater proportion in freshwater and soil systems (Fig. 1F). Comparing metagenomes with greater than 10 million genes with this metric indicated that *cld* was most enriched in environments such as oligotrophic rocks, sediment, and ice followed by oxic wastewater, surface freshwater, and dryland soils. Within aquatic environments, *cld* appears least frequently where chlorine is most concentrated: estuary, ocean, and hypersaline waters. The shared features of environments where *cld* is the highest proportion of coding genes is that they are predominantly oxic; many are also exposed to high amounts of sunlight [39, 40]. The broad distribution of the *cld* gene in genomes and metagenomes indicates chlorite is experienced by diverse microorganisms in various habitats.

### Evolution of Cld

**Fig. 2:**
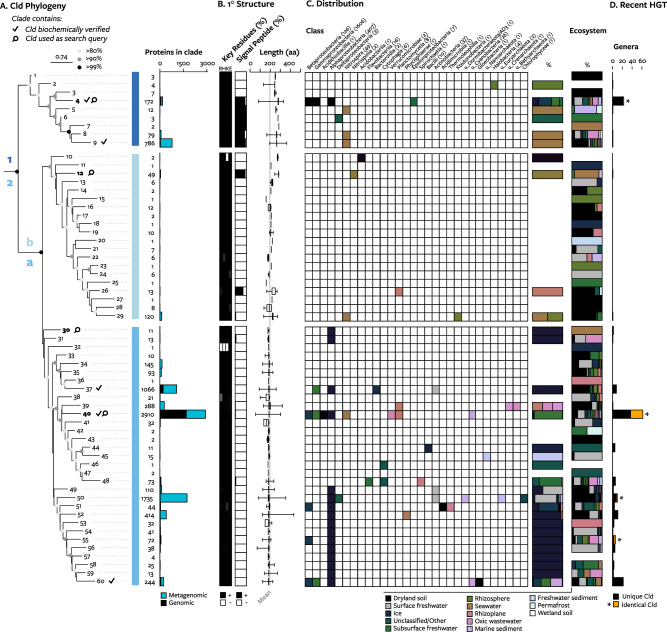
The phylogeny of Cld proteins and attributes of each lineage. **A** A maximum likelihood phylogenetic tree of Cld, with clades formed by phylogenetic distance and node support values indicated by color. Clades containing biochemically verified Cld proteins are indicated by a checkmark, and clades containing sequences used in the initial search are indicated by a magnifying glass. Major lineages of Cld are demarcated by dashed lines. The number of Cld per clade is listed at right and represented in a barplot by whether the source is genomic and metagenomic. **B** The primary structure of each clade. The proportion of each Cld clade computationally identified to have each key residue from 0% (white) to 100% (black) (left); the proportion of the clade computationally predicted to have a signal peptide (black) for export to the periplasm (center); and length of proteins in the clade represented as a boxplot where the box represents the interquartile range, whiskers represent maximum and minimum values, and the gray line represents the mean of all Cld (right). **C** Detection of Cld in each taxonomic class indicated by filled squares (left). Stacked barplots represent the proportion of each Cld clade in each environment (left) or each taxonomic class (right). **D** A measure of recent horizontal gene transfer: the number of genera found in each clade, with genera that can share an identical copy of Cld colored orange.

After its initial evolution from non-Cld proteins (Fig. 1A), Cld has continued to evolve. To better understand the history of Cld, a phylogeny was built from 8924 total Cld proteins, summarized as 60 clades formed by branch distance (Fig. 2). About half the diversity of Cld (29 clades) was constituted entirely of metagenomic sequences (Fig. 2A). Just one clade contains all Cld found in previously described bacteria that perform dissimilatory perchlorate or chlorate reduction (clade 4). Many of the largest clades include Cld proteins with biochemically verified chlorite: O_2_ lyase activity, and the key residues for activity were conserved in all clades except clade 38 (*n* = 21) and the under-sampled clades 10 (*n* = 2) and 32 (*n* = 1) (Fig. 2B), indicating the conservation of activity through geologic time.

The expanded phylogeny revises the previous understanding of the vertical evolution of Cld, wherein lineage 1 Cld are larger, periplasmic proteins and lineage 2 Cld are smaller, cytoplasmic proteins [23, 41]. First, tree topology showed two distinct, diverse, strongly supported (>99% bootstraps) sublineages within lineage 2 Cld, which we term lineage 2a and 2b. The only cultivated microorganism with lineage 2b Cld is *Nitrospina gracilis* [42]. Lineage 2b proteins are an intermediate length of 229 amino acids, and considerable variation in protein size was observed within the shorter lineage 2a Cld (Fig. 2B): 4% of Cld had larger (>20 aa) N- and C-terminal extensions that could either be artifacts of protein prediction or fusion proteins that augment the function of Cld [43]. Second, signal peptide prediction suggested that the more basal branching clades of group 1 Cld are not periplasmic, while two clades of lineage 2b Cld are periplasmic and a small number of Cld from various lineage 2a clades are periplasmic (Fig. 2B). The acquisition of peptide signals for export by Cld in many lineages indicates periodic selection for the degradation of extracellular chlorite.

Horizontal gene transfer of Cld across evolutionary time is evident from its taxonomic distribution. A single Cld clade can be found in taxonomic groups spanning phyla or even domains of life. (Fig. 2C). Cld has also been subject to recent transfer between genera: multiple Cld clades consisted of Cld from different genera (Fig. 2D). Alone, this metric reflects the combined signal of vertical and horizontal inheritance, but a detailed view shows that horizontal inheritance is a large component. For example, within clade 4, there are two instances where Cld from (per)chlorate-reducing proteobacteria appeared to have been acquired by nitrite-oxidizing *Nitrotoga* (Supplemental Fig. 2) [44]. Representing the most recent horizontal gene transfer: genomes from different genera possessed identical Cld proteins (Fig. 2D). In one case the same Cld protein (WP_011514928.1) was found in genomes from 18 genera. Anthropogenic sources of chlorine now appear to contribute to Cld’s evolution, evidenced by the first acquisition of Cld by an ammonia-oxidizing microorganism, *Nitrosomonas mobilis* Ms1, isolated from a wastewater treatment plant that used chlorine-based disinfectants (personal communication, Hirotsugu Fujitani) [45, 46]. The pattern of horizontal gene transfer suggests occasional yet strong selection for the ability to degrade chlorite. That can be reconciled with the rare conservation of Cld within phylogenetic groups (Fig. 1) by invoking a selective pressure for loss of Cld, possibly related to the heme requirement for this enzyme.

### Comparative genomics of Cld

**Fig. 3:**
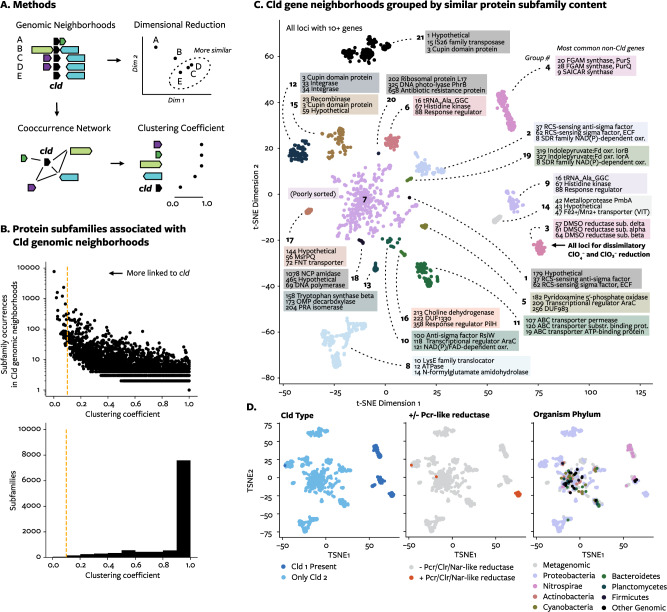
Statistical analysis of Cld genomic neighborhoods. **A** Schematic diagram explaining analyses. Genomic neighborhoods are compared using proteins clustered into protein subfamilies. In the gene-centric analysis, the co-occurrence of genes in different neighborhoods is used to construct a network, from which a clustering coefficient for each gene is derived. In the neighborhood-centric analysis, neighborhoods with more similar gene content are plotted through several dimensional reduction steps and clustered. **B** The distribution of protein subfamilies by their clustering coefficient, a measure of linkage to *cld*. The threshold value for defining “hits” is indicated. **C** Cld genomic neighborhoods colored by group, indicating thee top three most common proteins subfamilies in each group. Genomic neighborhoods that did not cluster into distinct groups are found in neighborhood group 7. **D** Cld genomic neighborhoods colored by the presence of group 1 Cld (left), by the presence or absence of reductases closely related to Pcr, Clr, and Nar (center), or by the phylum of the host microorganism (right).

Diverse microorganisms have experienced enough chlorite to select for the *cld* gene. Genes co-located with *cld* might be involved in chlorine redox biology. To identify genes correlated with *cld*, 61 215 proteins encoded in 8751 genomic neighborhoods of Cld (defined as the set of genes within 10 genes upstream or downstream of *cld*) were clustered into 11 081 protein subfamilies, whose distribution relative to each other can be described by the clustering coefficient (Methods, Fig. 3A). Subfamilies with a low clustering coefficient are found in many different types of genomic neighborhoods with Cld and, therefore, are more likely to have a function related to chlorine redox biology, rather than be co-located by chance. Only a small fraction of protein subfamilies in Cld genomic neighborhoods had a very low clustering coefficient (Fig. 3B, Table 1). Many of these proteins were already known to be related to chlorine redox biology, such as (per)chlorate reductases, oxidative stress response, signaling, and genetic mobility (e.g. tRNA as an insertion site) [47–49]. Additionally, since this work began, an alkylhydroperoxidase AhpD-like protein (subfamily 84), identified here as having a low clustering coefficient, was found to be the enzyme RcsA involved in hypochlorous acid degradation in *Pseudomonas aeruginosa* [50]. Therefore, this method identified true genetic associations with Cld.

**Table 1:** Genetic linkage of protein subfamilies to Cld.

SID	CC	Gene Product	Gene Function	Length (aa)	Count	Examples
cld_2	0.002	Chlorite dismutase, group 2	Chlorite degradation	188	7721	NP_773991.1, NP_924112.1
cld_1	0.019	Chlorite dismutase, group 1	Chlorite degradation	264	1069	WP_011288310.1, WP_013247962.1
3	0.026	Cupin domain-containing protein	Unknown	131	1591	WP_083761619.1, WP_011914407.1
19	0.027	ABC transporter ATP-binding protein	Transport	285	215	WP_008060994.1, WP_083842800.1
16	0.028	tRNA	Genetic mobility	-	260	
8	0.029	SDR family NAD(P)-dependent oxidoreductase	Unknown	258	409	NP_773983.1, WP_012078779.1
23	0.042	Site-specific DNA recombinase	Genetic mobility	193	201	WP_012435588.1, WP_001556711.1
80	0.050	Sigma-70 factor (ECF subfamily)	Regulation	217	70	NP_924104.1, WP_026605404.1
67	0.060	Signal transduction histidine kinase	Regulation	440	79	WP_003032875.1, WP_085107402.1
4	0.063	Transposase (IS66 family)	Genetic mobility	157	1202	WP_001515734.1
66	0.067	DNA-binding transcriptional regulator (LysR family)	Regulation	298	81	WP_020307717.1, WP_012435586.1
88	0.068	DNA-binding response regulator (OmpR family)	Regulation	226	65	WP_020177235.1, WP_001572351.1
53	0.071	DNA-binding response regulator (NarL/FixJ/NrtC family)	Regulation	188	86	WP_012078773.1, WP_007803317.1
34	0.072	Site-specific recombinase (XerD family)	Genetic mobility	321	139	WP_011914410.1
85	0.072	NADPH:quinone reductase-like Zn-dependent oxidoreductase	Unknown	332	68	WP_008175571.1, WP_007535313.1
1	0.073	Thermonuclease family protein	Genetic mobility	92	2334	WP_000046891.1
119	0.073	Signal transduction histidine kinase (NrtC family)	Regulation	650	51	WP_014235261.1, WP_066325839.1
15	0.074	Transposase (IS26 family)	Genetic mobility		269	WP_031992596.1, WP_038573166.1
33	0.074	Integrase	Genetic mobility	677	144	WP_011914409.1
60	0.074	DUF4113 domain-containing DNA polymerase	Genetic mobility	321	84	WP_080695106.1, WP_043755420.1
71	0.074	Translesion error-prone DNA polymerase V, umuD	DNA repair or salvage	134	77	WP_011914412.1, WP_043760689.1
99	0.075	DNA-binding transcriptional regulator (LysR family)	Regulation	296	57	WP_012078780.1, WP_012237598.1
64	0.077	Perchlorate/chlorate reductase, subunit beta	Perchlorate or chlorate reduction	323	81	WP_011288313.1, WP_029134664.1
57	0.081	Perchlorate/chlorate reductase, subunit delta	Perchlorate or chlorate reduction	212	84	WP_049758697.1, WP_037375986.1
70	0.081	TTT family transporter, receptor subunit	Transport	294	77	WP_009515871.1, WP_082751643.1
61	0.082	Perchlorate/chlorate reductase, subunit alpha	Perchlorate or chlorate reduction	904	83	WP_011288314.1, WP_037375984.1
55	0.082	Alpha/beta hydrolase family protein	Unknown	279	85	WP_009734230.1, WP_026779386.1
205	0.083	Enoyl-CoA hydratase/isomerase family protein	Unknown	228	33	WP_019497504.1, WP_083525184.1
77	0.084	Acetate-CoA ligase	Unknown	508	71	WP_009734228.1, WP_060979399.1
37	0.085	RCS-sensing anti-sigma factor, DUF1109 domain-containing	Oxidative stress response	208	132	WP_011288319.1, WP_003549388.1
54	0.085	Transposase (Tn3 family)	Genetic mobility	813	85	WP_012077404.1, WP_000124025.1
6	0.085	Gamma-glutamylcyclotransferase family protein	Oxidative stress response	114	665	NP_773992.1
116	0.085	Alpha/beta hydrolase family protein	Unknown	281	52	WP_040512021.1, WP_063988050.1
240	0.086	NAD(P)-dependent oxidoreductase	Unknown	310	29	WP_017285547.1, WP_020564915.1
62	0.088	RCS-sensing sigma factor	Regulation	177	83	WP_011288320.1, WP_003549387.1
93	0.091	NAD(P)/FAD oxidoreductase, glutathione sulfide reductase-like	Oxidative stress response	488	61	WP_008567178.1, WP_003158917.1
84	0.091	Alkylhydroperoxidase, AhpD-like	Oxidative stress response	182	68	WP_007535291.1, WP_036008191.1
97	0.091	Plasmid stabilization system toxin	Genetic mobility	97	59	WP_011342942.1, WP_094538652.1
107	0.091	Nitrate/sulfonate/bicarbonate ABC transporter permease	Transport	279	54	WP_007803303.1, WP_023100455.1
123	0.092	Peroxiredoxin	Oxidative stress response	165	50	WP_020096154.1, WP_058937083.1
95	0.092	Plasmid stabilization system antitoxin	Genetic mobility	91	60	WP_011342941.1, WP_011342941.1
11	0.092	DNA primase	Genetic mobility	604	310	NP_773990.1
128	0.093	Class I SAM-dependent methyltransferase	Unknown	228	48	WP_025297811.1, WP_083129309.1
22	0.093	DNA-binding transcriptional regulator (ArsR family)	Regulation	110	205	NP_773999.1, WP_103275825.1
14	0.095	N-formylglutamate amidohydrolase	Oxidative stress response	264	292	NP_773994.1
13	0.096	Adenylosuccinate lyase	DNA repair or salvage	425	304	WP_013247961.1, WP_033925750.1
162	0.098	Peptide-methionine (S)-S-oxide reductase	Oxidative stress response	203	38	WP_081614707.1, WP_003464967.1
110	0.098	DNA-binding transcriptional regulator (HxlR family)	Regulation	137	53	WP_015215298.1, WP_036002077.1
120	0.098	Nitrate/sulfonate/bicarbonate ABC transporter substrate-binding protein	Transport	422	51	WP_007803301.1, WP_023100454.1
74	0.099	DNA polymerase V, umuC	Genetic mobility	73	75	WP_011914411.1
177	0.100	Ferredoxin of nitrite reductase or dioxygenase	Unknown	110	36	WP_041756587.1, WP_005004323.1
129	0.100	Glutathione S-transferase family protein	Oxidative stress response	211	48	WP_015215307.1, WP_025659664.1
159	0.100	Peptide-methionine (R)-S-oxide reductase	Oxidative stress response	167	38	WP_019497506.1, WP_003464965.1

All subfamilies with a network clustering coefficient of less than 0.1 are shown. For Cld itself, the clustering coefficient should be interpreted to indicate the diversity of genomic contexts that Cld is found in; i.e. genomic neighborhoods of group 2 Cld occurs with have more genetic diversity than those of group 1 Cld. Columns: SID, subfamily ID; CC, clustering coefficient; Gene Product, description of the encoded protein or RNA; Gene Function, the predicted role for the gene in chlorine oxyanion biology; Length (aa), mean protein length; Count, total number of genes in the in the subfamily found in Cld genomic neighborhoods; and Examples, RefSeq accession for proteins in the subfamily from different classes of microorganisms.

Groups of highly similar genomic neighborhoods were defined by unsupervised clustering of neighborhoods with at least 10 genes (Fig. 3A). Neighborhood groups were distinguished by their most abundant non-Cld protein subfamilies (Fig. 3C) and recapitulated known differences (e.g. lineage 1 vs. 2 Cld, presence or absence of perchlorate and chlorate reductases, taxonomy) (Fig. 3D). (Lineage 1 and 2 Cld were only found together in the genomes of dissimilatory chlorate-reducing bacteria). Cld is found in a diverse set of genomic contexts: among the 20 neighborhood groups produced with this method, one single group contained all known genomic islands and composite transposons for respiratory perchlorate and chlorate reduction – the only natural pathways Cld has been confirmed participating in (Clark et al 2013, Melnyk and Coates 2015).

Clustering coefficients for protein subfamilies and grouping of neighborhoods were used to consider different aspects of chlorine biology: redox reactions, oxidative stress response, cellular transport, and chlorination. Because of their biochemistry, putative oxidoreductases with unknown function were among the most interesting proteins with genetic associations to Cld. Such oxidoreductases accounted for many of the subfamilies with the lowest clustering coefficients (Table 1): cupin domain-containing protein (subfamily 3), NADPH: quinone reductase-like Zn-dependent oxidoreductase (subfamily 85), and SDR family NAD(P)-dependent oxidoreductase (subfamily 8). Neighborhood-group 2 contained the SDR family NAD(P)-dependent oxidoreductase [51] as well as reactive chlorine-sensing regulatory elements that also had low clustering coefficients (subfamilies 37 and 62), further implicating a role this subfamily in reactive chlorine stress response. Fitness data for a protein in *Sphingomonas koreensis* DSMZ 15582 with 47% amino acid identity to a related SDR family NAD(P)-dependent oxidoreductase (subfamily 827, clustering coefficient 0.15) showed a deleterious effect when this protein was disrupted only in chlorite stress conditions or when glutamic acid was the carbon source [52]. The cupin domain protein was one of the most common subfamilies in the dataset, being found with Cld in 1487 genomes among 40 genera. In fact, encoded with transposases in neighborhood groups 12, 15, and 21, the cupin domain protein could be found in 90% of genomic neighborhoods with the most extreme form of horizontal gene transfer: encoding Cld proteins that are identical across different genera. The cupin domain protein had been suspected to have a role in reactive chlorine species response in (per)chlorate-reducing bacteria [53]. These data point to a far more common and important role for the cupin domain protein and other oxidoreductases in chlorine redox biology.

Cld genomic neighborhoods were searched for genes known to participate in the response to reactive chlorine species: methionine sulfoxide reductases, sulfur homeostasis proteins, protein chaperones, regulatory systems, and scavenging of reactive byproducts like peroxides, aldehydes, and glyoxals [6, 53, 54]. One or more of these genes could indeed be found in Cld neighborhood groups (Supplemental Data), and Cld was routinely found with methionine sulfoxide reductase systems (Supplemental Fig. 3). Responses to reactive chlorine species have largely been studied in laboratories; this is supporting evidence that use of these genes by microorganisms to respond to reactive chlorine species is not an experimental artifact but a natural phenomenon. Additionally, it provides evidence that chlorite is produced at a sufficient flux in the environment to contribute to oxidative damage in microorganisms.

The transport of chlorine oxyanions across the cellular membrane appeared to be a defining feature of two types of Cld genomic neighborhoods (Fig. 3C). No specific transporters for chlorine oxyanions are known. Neighborhood group 11 contained ABC transporter subunits, some of which are annotated as ATP-driven nitrate transporters. Such transporters could be involved in the transport of chlorate, a structural analogue of nitrate, an activity previously identified for nitrate transporters by genetic selection for chlorate resistance ((for example, see: [55])). Neighborhood group 17 was distinguished by a formate-nitrite transporter (FNT) family protein, an MsrP protein involved periplasmic reactive chlorine stress response (see below), and cytoplasmic Cld. As with nitrate and chlorate, formate (HCO_2_^−^) and nitrite (NO_2_^−^) are structural analogues of chlorite (ClO_2_^−^), and the potential for FNT family proteins to transport chlorite as well has been shown by the deleterious nature of FocA formate transporters and NirC nitrite transporters in chlorite stress conditions [52]. Curiously, the FNT-Cld-MsrP gene cluster belonged to metagenomic *Mycobacteria* found in seasonally low-oxygen lakes [56, 57]. The combination of a chlorite-permeable transporter and cytoplasmic Cld might act to import extracellular chlorite to be converted to oxygen inside the cell. Microorganisms benefitting from the production of oxygen by Cld is a trait thus far observed only in (per)chlorate-reducing bacteria or engineered strains [7, 58].

Chlorination and dechlorination are only known to be related to hypochlorous acid, not higher oxidation states of chlorine like chlorite. Relatively low clustering coefficients with Cld for two protein subfamilies suggested otherwise: non-heme chloroperoxidase (subfamily 122), which chlorinates organic molecules by producing hypochlorous acid, and a putative subfamily of haloacid dehalogenases (subfamily 172), which removes chlorine from organic molecules. Chlorite produces and may be produced by hypochlorous acid, which generates stable chlorinated products like chlorotyrosine [59]; perhaps microorganisms use dehalogenases to reverse that chlorination.

### Chlorite from chlorine reduction

**Fig. 4:**
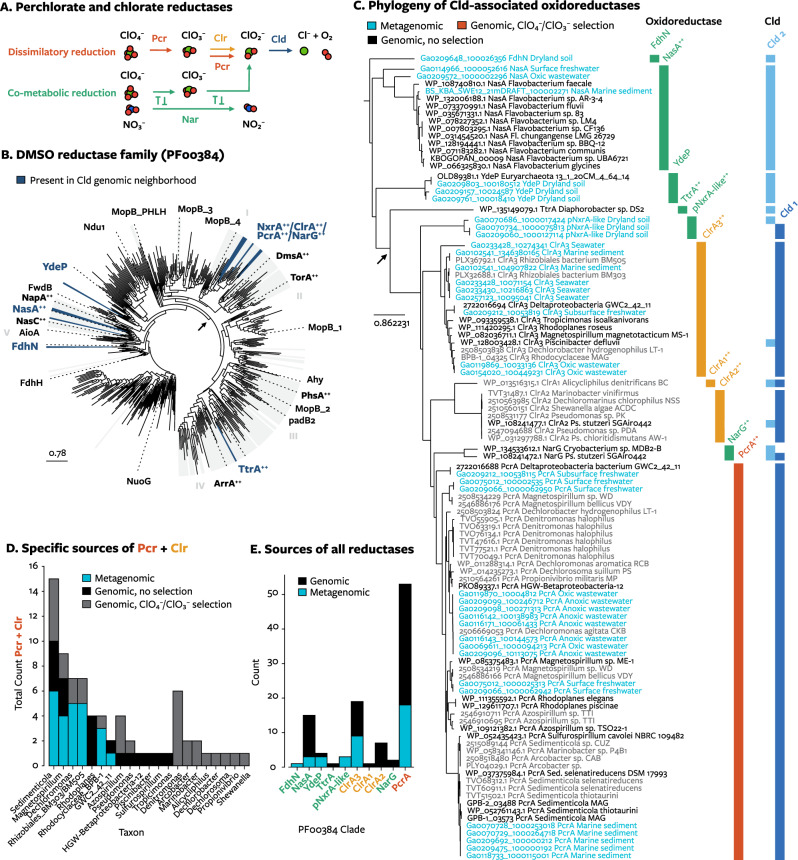
The distribution of Cld among possible perchlorate and chlorate reductases in the DMSO reductase family. Proteins with “++” have empirically described perchlorate or chlorate reductase activity. **A** The pathways by which a reductase can produce chlorite, which Cld degrades. Dissimilatory reduction occurs through perchlorate reductase (red, Pcr) or chlorate reductase (orange, Clr). Co-metabolic reduction (green) does not occur through a reductase specialized for perchlorate or chlorate reduction. An example is shown for nitrate reductase (Nar). **B** An unrooted maximum likelihood phylogenetic tree of representative proteins from the DMSO reductase family. Clades containing proteins from Cld genomic neighborhoods are highlighted in blue. **C** The same phylogenetic tree omitting all proteins not found with Cld. The arrow points to the same node as the arrow in panel **B**. Colors indicate their source (genomic or metagenomic). Labels at right indicate the type of protein and the lineages of Cld present in their genomic neighborhood. **D** The number of genomes per genus or other taxon with proteins from PcrA, ClrA1, ClrA2, or ClrA3, and whether or not the microorganisms were subjected to selection for those genes (i.e. providing perchlorate or chlorate as a sole respiratory electron acceptor). Metagenomic proteins were assigned to the closest genomic relative’s taxon. **E** The number of Cld-associated proteins in each clade of the DMSO reductase tree and whether they were obtained from genomes and metagenome-assembled genomes (black) or metagenomes (blue).

One source of chlorite experienced by microorganisms could be the co-metabolic reduction of perchlorate and chlorate. Comparative genomics can be used to evaluate this hypothesis: if an enzyme reduces perchlorate or chlorate to chlorite, Cld can provide a benefit by degrading chlorite, and the selective pressure to co-express the Cld with would lead to its genetic co-location with the reductase in some genomes (Fig. 4A). To test this hypothesis, Cld genomic neighborhoods were searched for enzymes in the DMSO reductase family of molybdopterin enzymes (Pfam 00384), which contains both the specialized perchlorate and chlorate reductases (Pcr, Clr) and the enzymes that inadvertently reduce perchlorate and chlorate [17].

A total of 105 proteins in the DMSO reductase family were found in Cld genomic neighborhoods. Many genomic neighborhoods contained cytoplasmic Cld and enzymes with documented in vitro (per)chlorate reductase activity (Fig. 4B–E): assimilatory nitrate reductases (NasA), a cytoplasmic dissimilatory nitrate reductase (NarG) [60], and a tetrathionate reductase (TtrA) [61]. Cld was also found on soil metagenome contigs with uncharacterized enzymes most similar to periplasmic nitrite oxidoreductases (pNxr) of nitrite-oxidizing bacteria (*Nitrospira* and *Nitrotoga*), anammox bacteria, and other microorganisms [44]. As it is found with either periplasmic or cytoplasmic Cld, this uncharacterized reductase is not likely to function in dissimilatory (per)chlorate reduction pathways, which are only known to occur in the periplasm [8]. The co-occurrence of Cld with formate dehydrogenase (FdhN) and an uncharacterized Fdh-like protein (YdeP) was unexpected but could be related to the structural similarity of chlorite and formate. The genetic association of Cld with diverse enzymes with co-metabolic (per)chlorate reductase activity indicates that co-metabolism contributes to perchlorate and chlorate reduction.

Most commonly, the reductases detected with Cld in metagenomes were not co-metabolic reductases but Pcr and the newly characterized group 3 Clr [62] (Fig. 4D, E). Most reductases were closely related to reductases from organisms selected for the ability to respire perchlorate or chlorate. The search also identified a metagenome-assembled genome, GWC2_42_11, assembled from a groundwater sediment metagenome [63], encoding phylogenetically divergent copies of both Pcr and group 3 Clr (Fig. 4C). Two genes for Cld from this microorganism are found in Cld clade 6, which share a more recent ancestor with nitrite-oxidizing *Nitrospira* (clade 5, clades 7–9) than perchlorate-reducing bacteria (clade 4) (Fig. 2). As a member of the class *Deltaproteobacteria* (phylum GWC2–55–46 in GTDB taxonomy), GWC2_42_11 is the most evolutionary distinct (per)chlorate-reducing bacterium identified to date, and its equally divergent reductases and Cld might help in understanding the earliest forms of perchlorate and chlorate respiration.

The ancient form of dissimilatory (per)chlorate reduction may have resembled co-metabolic (per)chlorate reduction by a microorganism with Cld. Cld has been shown to be inessential for removing any chlorite produced if habitats with sufficient amounts of reduced iron or inorganic sulfur species [64–66] (or with large populations of other microorganisms that can degrade chlorate or chlorite [16, 58]), allowing a scenario where chlorite dismutase could evolve after specialized (per)chlorate reductases. Yet the above results show that chlorite stress from co-metabolic (per)chlorate reduction is a common enough phenomenon that Cld has repeatedly evolved to be co-located with co-metabolic reductase in genomes. This is a contemporary example of how respiratory metabolisms for oxidized chlorine could have first arose from the association between chlorite dismutase and a co-metabolic reductase followed by specialization for respiratory perchlorate or chlorate reduction [67]. In that scenario, chlorite dismutase would have evolved before specialized (per)chlorate reductases.

### Chlorite from chlorine oxidation

**Fig. 5:**
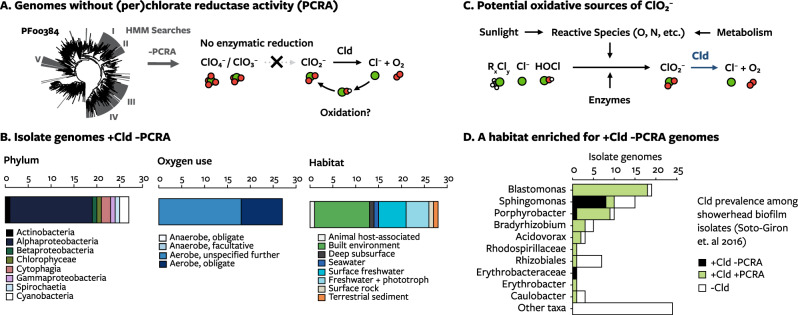
Genomes without respiratory and co-metabolic perchlorate/chlorate reductase activity (PCRA). **A** Profile-HMMs were used to find isolated microorganisms without enzymes from the broad parts of the DMSO reductase family that might have (per)chlorate reductase activity. These microorganisms may experience chlorite produced from oxidative chemistry. **B** The number of isolate genomes with respiratory or co-metabolic reductases grouped by phylum, relationship with oxygen, and the habitat they were isolated from. **C** Pathways discussed in the text as having the potential to generate chlorite from lower oxidation states of chlorine. **D** Several microorganisms without PCRA were isolated from a showerhead biofilm communities exposed to chlorine residuals present in drinking water. Bars indicate the number of microorganisms isolated from that community with or without Cld or enzymes with putative (per)chlorate reductase activity.

The oxidation of chlorine to chlorite is another possible source, other than co-metabolic reduction of (per)chlorate or chemical reduction of chlorate [68], of chlorite in oxic habitats. If it occurs, Cld should be present in microorganisms unable to co-metabolically reduce (per)chlorate. Using profile-HMMs representing the broad parts of the DMSO reductase family phylogeny that have perchlorate or chlorate reductase activity (PCRA) (Fig. 4A), we identified genomes with Cld that do not have enzymes that reduce perchlorate or chlorate (Fig. 5A). Despite the commonality of such enzymes as assimilatory nitrate reductases, this search identified 27 putative “non-(per)chlorate reducers” among isolate genomes (Supplementary Table 1). These strains represent 6 of the 19 phyla with Cld and 15 of 151 genera (Fig. 5B). All were reported aerobes (Fig. 5B). They were isolated from diverse habitats, often characterized by high sunlight (lakes and ponds, desert rocks and sediments, growing with diatoms, cyanobacteria, or mosses) or by high amounts of reactive chlorine species (human body, wastewater treatment plant, swimming pool, showerhead biofilm) (Fig. 5B). Therefore, the known mechanisms for the enzymatic reduction of chlorate and perchlorate appeared insufficient to explain the prevalence of chlorite and chlorite-degrading microorganisms.

One plausible mechanism of oxidative chlorite formation is oxidative chemistry (Fig. 5C). Habitats with high sunlight are very oxidizing due to the combined effects of oxygenic phototrophy and UV photochemistry [69, 70]. Several non-(per)chlorate reducers were isolated from high sunlight habitats. UV tolerance genes were present in Cld genomic neighborhoods from several bacteria from high sunlight habitats. A putative deoxyribodipyrimidine photo-lyase, which is a light-activated protein that repairs UV-damaged DNA, is encoded in 21 Cld genomic neighborhoods and several different neighborhood groups (groups 2, 20, and 7), such as one in a betaprotebacterium in culture with *Leptolyngbya glacialis* TM1FOS73 (GCA_003242045.1) [71]. In a sunlight photobioreactor metagenome, *cld* is found in four of 34 MAGs (UBA7691, UBA7681, UBA7678, UBA7677), including a *Planctomycetaceae* bacterium that has periplasmic group 2a Cld encoded near carotenoid biosynthesis genes for limiting UV photodamage [72]. The production of chlorite from oxidative chemistry might also explain the presence of Cld in the nitrite-oxidizing bacteria (Fig. 5C). Like the products of photochemistry such as hydrogen peroxide (H_2_O_2_, *E*^*0*^*’* + 1.32 V) [73], the reactive nitrogen species peroxynitrite (ONOO^−^, *E*^*0*^*’* + 1.3 V) has a high enough reduction potential to oxidize hypochlorous acid to chlorite (*E*^*0*^*’* + 1.26 V) [74, 75].

Another plausible mechanism of chlorine oxidation is the biochemical oxidation of hypochlorous acid to chlorite (Fig. 5C). An enzyme that oxidizes hypochlorous acid to chlorite would be a major fitness benefit to microorganisms with Cld, ultimately yielding harmless chloride and oxygen. Oxidation of hypochlorous acid to chlorite only requires transfer of 2 electrons and produces a less reactive product. An analogous system would be nitric oxide dioxygenase, which uses oxygen to oxidize nitric oxide to less-toxic nitrate [76]. Instead of spending cellular reducing equivalents to reduce hypochlorous acid or repair oxidative damage, the enzymatic oxidation of hypochlorous acid might produce reducing equivalents. Furthermore, the removal of hypochlorous acid would limit the inhibition of Cld by hypochlorous acid [77]. Thus, the enzymatic oxidation of hypochlorous acid to chlorite would pose major selective benefits.

Experimental support for this capability would be the enrichment of microorganisms with Cld in habitats with high hypochlorous acid. One such real-word setting appeared to be a drinking water distribution system in which 47 of 89 strains isolated from a showerhead biofilm encoded Cld, and 10 were non-(per)chlorate reducers (Fig. 5D) [78, 79]. This demonstrates a strong selection for Cld within the microbial community by the chlorine residuals present in the water. The water distribution system was expected to contain 0.8 mg/liter free residual chlorine form of hypochlorous acid and hypochlorite residuals; however, it is unclear if chlorine dioxide (ClO_2_) was used in water treatment and produced chlorite residuals (personal communication, Jorge Santo-Domingo). Except for this uncertainty, this system would meet the criteria of a habitat that selects for the ability to degrade chlorite due to only high hypochlorous acid exposure. Enzymatic oxidation of hypochlorous acid to chlorite remains an unproven hypothesis.

### A holistic model for chlorine redox biology

**Fig. 6:**
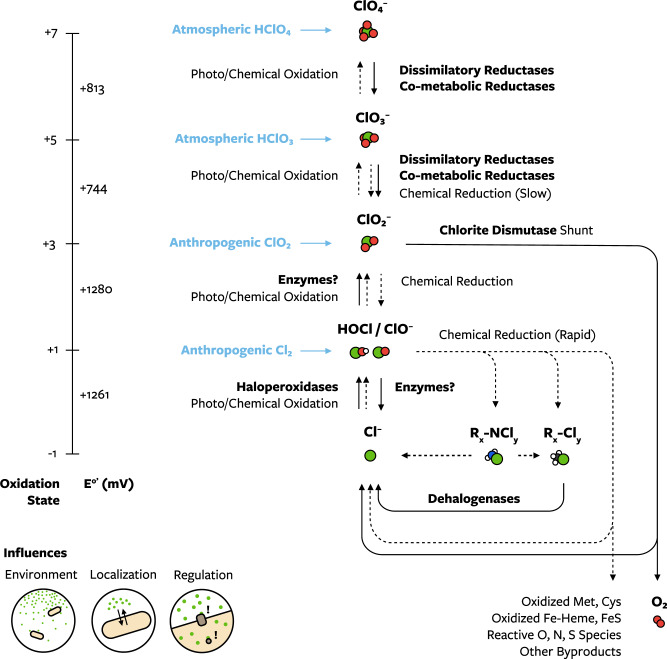
A model for biological chlorine reduction and oxidation reactions occurring within biological habitats. Biological (solid arrows, bold) and chemical or photochemical (dashed arrows) reduction and oxidation reactions of chlorine that occur, in aqueous solution or within microbial cells. Halogenases, the primary source of organochlorine, are omitted for simplicity. Other oxidized chlorine species can be external inputs into biological systems (blue). Vertical position corresponds to changes in chlorine’s formal oxidation state and reduction potential at standard conditions (pH 7, 25 °C, solutes at 1 M) in millivolts (gray). Additional factors that influence chlorine redox biology but do not perform redox reactions are shown: habitat (pH, redox potential, etc.), cellular composition including transporters, and cellular signaling and responses. Abbreviations: R-N_x_Cl_y_ organic and inorganic chloramines, R_x_-Cl_y_ organochlorine, ClO_2_ chlorine dioxide, Cl_2_ molecular chlorine.

The different biological processes that involve Cld suggest that the biology of chlorine reduction and oxidation should be considered as a single, bidirectional pathway (Fig. 6). In this model, based on the above data and previous studies, microorganisms in many habitats can experience any chlorine oxyanion and some anthropogenic oxidized chlorine species (Cl_2_, ClO_2_, NH_2_Cl, NHCl_2_, and NCl_3_) [80, 81]. Transporters allow oxidized chlorine species to enter cells, where the molecules or their byproducts may be sensed and lead to changes in gene regulation. Oxidized chlorine species have a propensity to be reduced in cells to the next lower oxidation state. Chlorite is produced in microorganisms with enzymes that can reduce perchlorate and chlorate through metabolism or co-metabolism. Cld can be considered a shunt in the reductive pathway that, when present, prevents the formation of hypochlorous acid and produces beneficial oxygen. Any hypochlorous acid formed reacts rapidly with biomolecules, producing a combination of chloride, chlorinated carbon and nitrogen, and oxidized byproducts [2, 3, 5, 82]. Cells degrade hypochlorous acid and respond to damage caused by it. A hypothetical possibility is that microorganisms detoxify hypochlorous acid by oxidizing hypochlorous acid to chlorite and using the chlorite dismutase shunt to degrade chlorite. In oxidizing settings, chlorite can be further oxidized (photo)chemically to chlorate or perchlorate, which are also deposited into habitats from atmosphere. The relatively stable end products of this bidirectional cycle are perchlorate and chloride. These compounds are only as inert, however, as the surrounding chemistry and biology allow.

## Conclusions

Cld, a biomarker for chlorite once thought unique to anaerobic perchlorate- and chlorate-reducing bacteria, is found in various microorganisms from both oxic and anoxic microbial habitats. This distribution suggests microorganisms experience significant enough amounts of chlorite in the environment to acquire Cld. The sources of chlorite are the dissimilatory reduction of (per)chlorate but also the co-metabolic reduction of (per)chlorate and, genomics suggests, the oxidation of chlorine’s lower oxidation states. That Cld participates in these pathways and in general response to reactive chlorine species justifies a model wherein oxidized chlorine species are part of a continuous, bidirectional biological pathway. Cld is subject to intermittent selection for its gain and loss, highlighting how much remains to be learned about the concentrations and fluxes of oxidized chlorine species in different environments. The expansive inventory of genes associated with Cld-encoding loci identified here provides targets for subsequent research in the biology of oxidized chlorine from regulation, transport, and repair to direct enzymatic action on chlorine-containing molecules.

## Methods

### Identification of chlorite dismutase (Cld)

A maximum likelihood phylogenetic tree of the protein family containing Cld was constructed using FastTree v. 2.1.9 with default settings from the Pfam 06778 alignment of representative proteomes, at the 15% comembership threshold to limit the number of redundant proteins [83–85]. The presence of key residues for Cld activity were identified by comparing the positions in the alignment corresponding to the distal heme arginine (R127) and proximal heme lysine (K92), histidine (H114), and glutamic acid (E167) in *Nitrobacter winogradskyii* Nb-255 [29]. Cld and non-Cld proteins were annotated on a precomputed bacterial tree of life [86]. Cld was identified through BLASTP search of genomes deposited in NCBI and genomes and metagenomes deposited in JGI [87–89] and confirmed with profile-hidden Markov models built from the two major lineages of Cld from Pfam 06778 [90]. All data were acquired prior to 2020. See supplementary information for additional methods.

### Phylogenetics

For the phylogeny of Cld, proteins were aligned using MUSCLE v3.8.1551 with default settings [91] and built into a maximum likelihood phylogenetic tree using FastTree with default settings [85]. The Python package ETE v. 3 was used to plot trees and to form clades of proteins at trees nodes in which the average distance to a protein was less than a selected value [92]. N-terminal and C-terminal extensions were defined as amino acids in the alignment beyond the positions within which the average Cld protein had amino acids. Signal peptides were assigned using SignalP v. 5 with default settings, accepting a positive result from any type of organism [93]. See supplementary information for additional methods.

### Comparative genomics

Genes within +/− 10 positions of *cld* on the same contig were defined as part of the Cld genomic neighborhood. Proteins from Cld genomic neighborhoods were clustered into subfamilies using MMSEQs v.7–4e23d set to a coverage of 0.5 and an E-value of 0.001 [94]. The Python package networkx was used to compute the clustering coefficient for each node. This is a simple statistic for gene linkage to *cld* obtained by representing each subfamily as a node and each connection between subfamilies found in the same genomic neighborhood as edges in a network. The clustering coefficient for a node equals the number of edges between a node’s neighbors divided by the total number of edges possible between a node’s neighbors (for more information, see supplementary information).

Genomic neighborhoods with 10+ genes were grouped by similar gene content using unsupervised machine learning methods in the Python package SciKit-learn. The features were the presence [1] or absence (0) of each subfamily in the neighborhood. An initial dimensional reduction was performed with Principle Components Analysis, and the resulting 50 dimensions were subjected to t-Distributed Stochastic Neighbor Embedding (t-SNE) with a perplexity of 50 and 5000 iterations. Neighborhoods were then grouped by proximity (maximum distance of 4 units in the two t-SNE dimensions) with the Density-Based Spatial Clustering of Applications with Noise (DBSCAN) algorithm.

## Supplementary information

Supplementary Information

SI Table 1

## Data Availability

Supplementary data are available on FigShare and include: Supplementary Data 1, information on genes and genomes used in this work including accessions, taxonomy, subfamily assignments, etc. (10.6084/m9.figshare.16978561); Supplementary Data 2, information on subfamilies and their clustering coefficients (10.6084/m9.figshare.16980601); protein sequences found in Cld genomic neighborhoods (10.6084/m9.figshare.16980613); a phylogenetic tree and alignments for Cld (10.6084/m9.figshare.16982077); and profile-HMMs to identify key proteins for perchlorate, chlorate, and chlorite biology (10.6084/m9.figshare.19836151).
