# Supplementary material for: Chlorine redox chemistry is widespread in microbiology
Source: ISME J. 2022 Oct 6;17(1):70–83. doi: 10.1038/s41396-022-01317-5 (PMC9751292; doi:10.1038/s41396-022-01317-5)
Supplement: Supplementary file 1 — Supplementary Information [file 41396_2022_1317_MOESM1_ESM.pdf]

Supplementary Figure 1
Maximum likelihood phylogeny of Cld lineage 2.

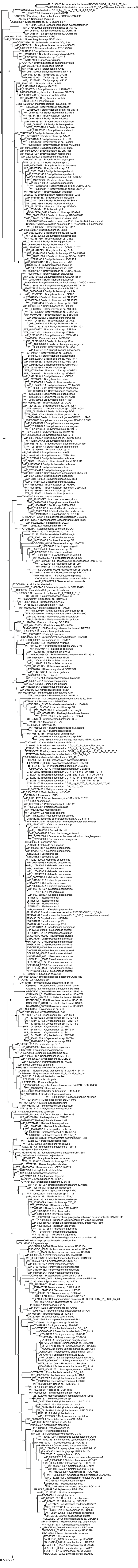

Supplementary Figure 2

Maximum likelihood tree of Cld lineage 1.

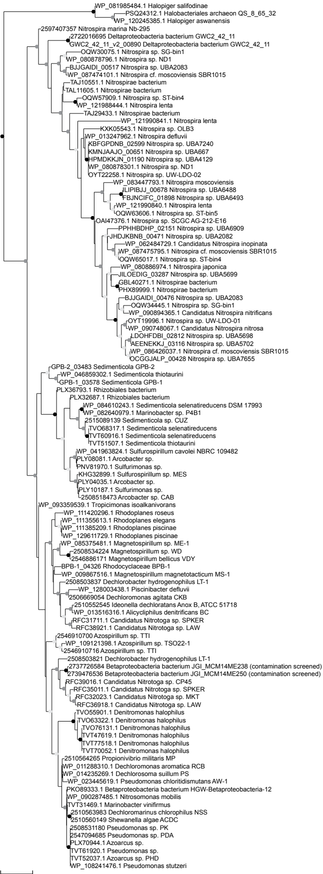

Supplementary Figure 3

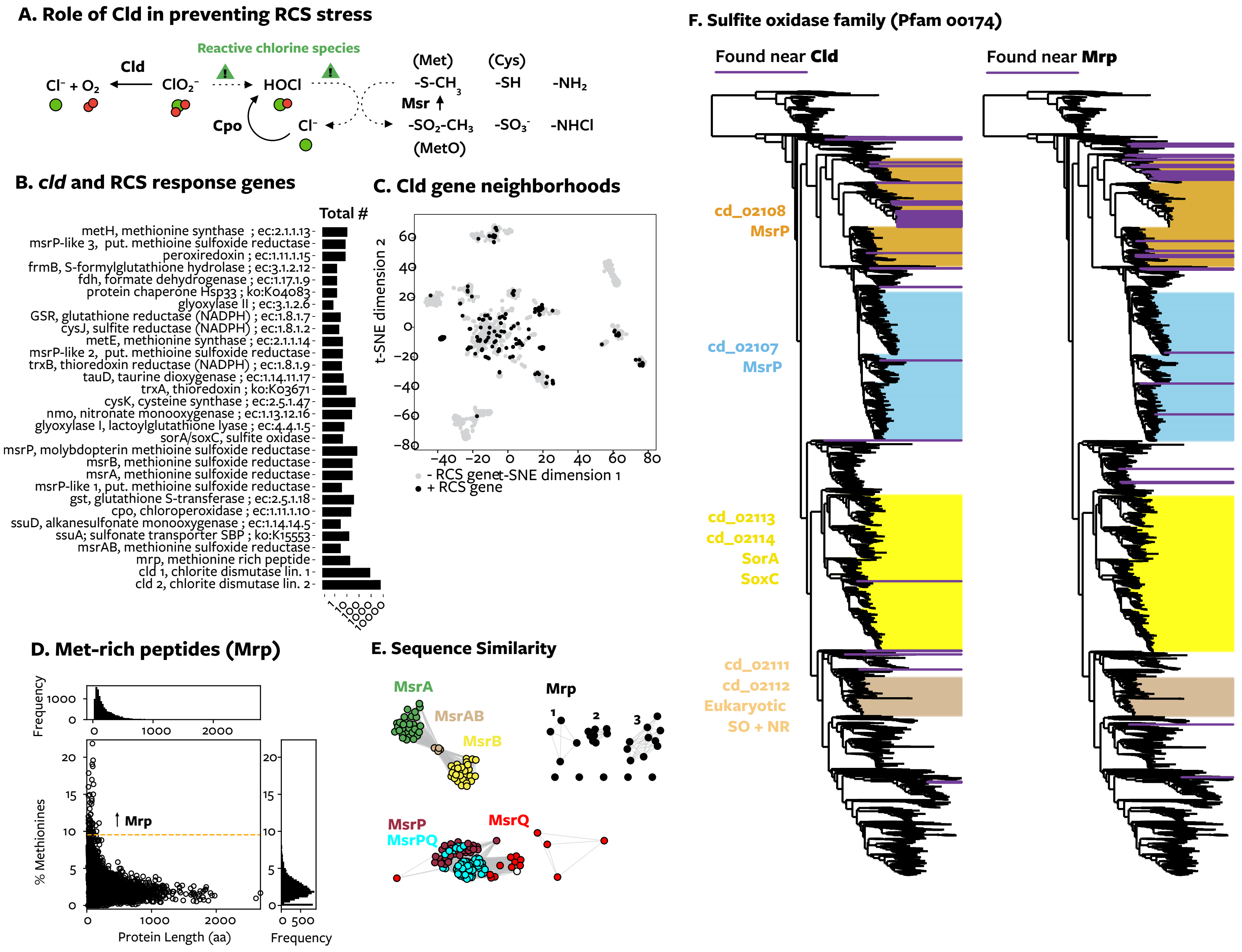

Description of Supplementary Figure 3

This figure shows the association of Cld with genes for reactive chlorine species response.

Panel A is a schematic diagram illustrating why Cld, having chlorite as a substrate, would be associated with HOCl and genes for repairing HOCl-mediated oxidative damage: HOCl is produced from the reduction of chlorite and may be a major source of oxidative damage in chlorite reduction. Panel B shows the frequency of reactive chlorine species response genes found with Cld, in number of genes detected. Panel C shows the Cld genomic neighborhoods including reactive chlorine species response genes. Together these data show how widespread known genes for reactive chlorine species response are within Cld genomic neighborhoods.

An important component of reactive chlorine species response in need of further definition is the methionine sulfoxide reductase system. Hypochlorous acid most rapidly and specifically oxidizes sulfur atoms in amino acids, converting methionine to methionine sulfoxide and progressively oxidizing cysteine to sulfenic acid (1, 2). Methionine is regenerated by methionine sulfoxide reductases: MsrA, MsrB, and MsrP (formerly YedY) (3-5). The importance of MsrP reductases is evident from their being the most common beneficial genes in chlorite stress conditions (6), but MsrP and Mrp – a methionine rich peptides that scavenges hypochlorous acid and chlorite is sometimes co-expressed with MsrP (5) – are poorly defined.

New putative Mrp were defined by fitting a normal distribution to the mean Met content of all protein subfamilies in Cld genomic neighborhoods and selecting small proteins with exceptional Met content ($p < 0.00001$, >8.7% Met) found with MsrA, MsrB, or MsrP. Panel D shows the distribution of the mean methionine content in protein subfamilies (% methionine) used to define methionine rich peptides (Mrp). The phylogenetic relationships between proteins in the methionine sulfoxide reductase system were investigated using a network analysis, shown in panel E. Unlike Msr enzymes, Mrp formed multiple distinct groups that had no significant sequence similarity to one another, indicating these short HOCl-scavenging peptides might have evolved independently multiple times. These peptides may be like other short peptides that evolved *de novo*, from noncoding sequences (7).

MsrP methionine sulfoxide reductases belong to a larger family of proteins, the sulfite oxidase family of molybdopterin enzymes (Pfam 00174), which has several conserved domains of unknown function. To define which proteins in the sulfite oxidase family act as methionine sulfoxide reductases, proteins found with Cld were placed into a maximum likelihood phylogenetic tree of Pfam 00174 including proteins from representative proteomes. Extending a previous analysis using Mrp (5), nodes in the tree were further annotated if Mrp, a substrate of MsrP, was found within 5 genes of the molybdopterin domain gene. Panel F shows a version of the tree with each annotation: proteins found with Cld (left tree) or Mrp (right tree) are highlighted blue, and clades are highly by their conserved domain annotation. These genomic signatures of activity span the breadth of both conserved domains that contain characterized methionine sulfoxide reductases: MsrP from *Azospira suillum* PS (cd_02108) and MsrP from *Escherichia coli* and *Rhodobacter sphaeroides* (cd_02107) (4, 5, 8). The occurrence of Cld and

Mrp were found in other clades that could be methionine sulfoxide reductase or are traditional sulfite oxidases. This could be spurious, but a potential functional link between sulfite oxidases and Cld may be related to the involvement of sulfite oxidases in recycling sulfur oxidized by reactive oxidants (9).

Supplemental Methods

Identification of chlorite dismutase (Cld)

BLASTP was used to identify Cld in genomes. with RefSeq preferred. BLASTP was used to identify metagenomic Cld in JGI IMG/M among the largest metagenomes consisting of 90% of proteins in each "Ecosystem Category." Metagenome-assembled genomes in the Uncultivated Bacteria and Archaea (UBA) dataset were searched directly with profile-HMMs (10). The query proteins (accession, bitscore threshold) used in the BLASTP search were Cld from Dechloromonas agitata (AAM92878.1, 125), Nitrospina gracilis (WP_005007586.1, 150), Sandarakinorhabdus cyanobacteriorum (WP_094475098.1, 60), and Pseudomonas stutzeri (WP_026084028.1, 100). All Cld identified with BLASTP were confirmed with profile-HMMs using HMMER version 3.2.1 and bitscore thresholds of 90 (all Cld), 100 (lineage 1 Cld), and 90 (lineage 2 Cld).

Genomic data and metadata were downloaded and processed using custom scripts. No criteria were used to exclude genomes, but metagenome-assembled genomes discussed in the results were manually evaluated for whether the contig containing Cld was correctly assigned to that genome.

The fraction of a taxonomic group encoding Cld was determined by comparing the number of RefSeq genomes with the *cld* gene to the total number of RefSeq genomes available within each taxonomic group (https://github.com/kblin/ncbi-genome-download). The detection of Cld in different environments was compared using the number of *cld* copies per million total coding domain sequences (CDS) obtained from IMG/M metagenome metadata. Due to inconsistent definitions of environments in IMG/M, metadata were used to assign each metagenome to a custom environmental category.

Phylogenetics

Proteins in the DMSO reductase family of molybdopterin enzymes, which might function as perchlorate and chlorate reductases, were identified using a profile-HMM built from the seed alignment of Pfam 00384 and a bitscore threshold of 200 (11, 12). A maximum likelihood phylogenetic tree was constructed from those proteins encoded near *cld* and a curated set of proteins from Pfam 00384 proteins in representative proteomes at the 15% comembership threshold. The curated set of proteins were constructed as follows: incomplete proteins were excluded using a size threshold of 300 amino acids, the size of dataset was reduced while maintaining diversity by clustering proteins at 50% amino acid identity using CD-HIT. Only positions in the alignment where a majority of proteins had residues were kept. The tree was constructed, plotted, and grouped into clades as above.

Phylogenetics of the chlorite dismutase protein family are described in the main text.

Comparative genomics

In select instances, genes were compared to fitness experiments using chlorite and chlorate on the Fitness Browser (fit.genomics.lbl.gov) described in Price et. al 2018. That paper describes genetics experiments comparing the fitness of individuals within pooled transposon insertion libraries (i.e. populations where different cells have different genes disrupted by transposons) between different conditions. Chlorite and chlorate would be considered stress conditions, and individuals in the population do relatively worse if a transposon disrupts a gene important for mitigating that stress.

The main text describes the clustering coefficient, a property of a node in networks. The clustering coefficients is defined as the fraction of possible edges between a node's neighbors that have been realized. For example, a node is connected to 3 neighbor nodes has only 3 possible total edges between the neighbors; if there is only 1 edge between its neighbor nodes, the node's clustering coefficient equals 1 divided by 3. For a gene in a Cld genomic neighborhood, the clustering coefficient quantifies how many different sets of genes the gene is found with. With enough observations, this statistic easily differentiates a gene always found with the same genes (e.g. a large biosynthetic operon) from a gene found repeatedly with various genes (e.g. a transposase).

Additional information about subfamily notation: The two major lineages of Cld were manually split into separate subfamilies. All other subfamilies were numbered in order of their size in this dataset.

Description of Supplementary Data

Supplementary Data 1 contains information on genes found within Cld genomic neighborhoods, defined as genes within +/- 10 genes of Cld. This data, with protein sequences, should enable the reproduction of results reported in this paper. Key information includes the accessions and coordinates of genes, annotation of the gene, the taxonomic assignment of genomes or scaffolds, clustering of proteins into groups, and clustering of neighborhoods into groups. Additional data on groups of proteins is found in Supplementary Data 2

Supplementary Data 2 contains descriptions of protein subfamilies found in Cld genomic neighborhoods. Protein subfamilies are the result of clustering proteins together based on homology and were used to compare the composition of Cld genomic neighborhoods from different organisms. The clustering coefficient can be used to identify sequences with the strongest association to Cld and therefore the likeliest involved in the biology of oxidized chlorine: lower clustering coefficients indicate a stronger association.

cld-genomic-neighborhood-proteins.faa.gz is a gzipped FASTA file containing all protein sequences identified within Cld genomic neighborhoods. This data and Supplementary Data 1 would enable reproduction of this analysis.

cld-tree.tar.gz contains the Cld phylogenetic tree for this dataset and the sequences and alignments used to build the tree.

profile-hmms.tar.gz contains the profile hidden Markov models used in this work.

References for Supplement

1. Gray MJ, Wholey W-Y, Jakob U. Bacterial responses to reactive chlorine species. Annual Review of Microbiology. 2013;67:141-60.
2. Winterbourn CC. Reconciling the chemistry and biology of reactive oxygen species. Nature Chemical Biology. 2008;4:278-86.
3. Ezraty B, Gennaris A, Barras F, Collet J-F. Oxidative stress, protein damage and repair in bacteria. Nature Reviews Microbiology. 2017;15:385.
4. Gennaris A, Ezraty B, Henry C, Agrebi R, Vergnes A, Oheix E, et al. Repairing oxidized proteins in the bacterial envelope using respiratory chain electrons. Nature. 2015;528(7582):409-12.
5. Melnyk RA, Youngblut MD, Clark IC, Carlson HK, Wetmore KM, Price MN, et al. Novel Mechanism for Scavenging of Hypochlorite Involving a Periplasmic Methionine-Rich Peptide and Methionine Sulfoxide Reductase. mBio. 2015;6:e00233-15.
6. Price MN, Wetmore KM, Waters RJ, Callaghan M, Ray J, Liu H, et al. Mutant phenotypes for thousands of bacterial genes of unknown function. Nature. 2018;557(7706):503-9.
7. Knopp M, Gudmundsdottir JSN, Tobias; , König F, Warsi O, Rajer F, Ädelroth P, et al. De Novo Emergence of Peptides That Confer Antibiotic Resistance. 2019;10(3):e00837-19.
8. Tarrago L, Grosse S, Siponen MI, Lemaire D, Alonso B, Miotello G, et al. Rhodobacter sphaeroides methionine sulfoxide reductase P reduces R- and S-diastereomers of methionine sulfoxide from a broad-spectrum of protein substrates. Biochem J. 2018;475(23):3779-95.
9. Kappler U, Schwarz G. The Sulfite Oxidase Family of Molybdenum Enzymes. 2017.
10. Parks DH, Rinke C, Chuvochina M, Chaumeil P-A, Woodcroft BJ, Evans PN, et al. Recovery of nearly 8,000 metagenome-assembled genomes substantially expands the tree of life. Nature Microbiology. 2017;2:1533-42.
11. Finn RD, Clements J, Arndt W, Miller BL, Wheeler TJ, Schreiber F, et al. HMMER web server: 2015 update. Nucleic Acids Research. 2015:1-9.
12. El-Gebali S, Mistry J, Bateman A, Eddy SR, Luciani A, Potter SC, et al. The Pfam protein families database in 2019. Nucleic Acids Research. 2018;47(D1):D427-D32.
