# Supplementary material for: Chlorine redox chemistry is widespread in microbiology
Source: ISME J. 2022 Oct 6;17(1):70–83. doi: 10.1038/s41396-022-01317-5 (PMC9751292; doi:10.1038/s41396-022-01317-5)
Supplement: Supplementary file 2 — SI Table 1 [file 41396_2022_1317_MOESM2_ESM.pdf]

SupplementaryTable-1

Phylum	Organism	Oxygen Requirement	Isolation Source	Environment Type	Genome Accession	Reference (DOI/Biosample)
**Actinobacteria**	Modestobacter sp. DSM 44400	Unspecified aerobe	Yellow mineral on sandstone in Antarctica	Surface rock	GCF_900107175.1	SAMN05661080
**Bacteroidetes**	Algoriphagus sp. DSL-12	Obligate aerobe	Seawater at junction with freshwater lake	Seawater	GCF_003259255.1	10.1007/s10482-019-01280-w
**Bacteroidetes**	Hymenobacter roseosalivarius DSM 11622	Obligate aerobe	Sediment from Antarctic Dry Valleys	Terrestrial sediment	GCF_900176135.1	10.1016/S0723-2020(98)80047-7
**Chlorophyta**	Monoraphidium neglectum	Oxygenic phototroph	Freshwater	Surface freshwater (phototroph-associated)	GCF_000611645.1	10.1186/1471-2164-14-926
**Cyanobacteria**	Anabaena cylindrica PCC 7122	Obligate aerobe	Pond water	Surface freshwater	GCF_000317695.1	10.1099/00221287-111-1-1
**Cyanobacteria**	Neosynechococcus sphagnicola sy1	Unspecified aerobe	Endophyte of Sphagnum in peat bog	Surface freshwater (phototroph-associated)	GCF_000775285.1	10.11646/phytotaxa.170.1.3
**Proteobacteria**	Acinetobacter baumannii OIFC074	Obligate aerobe	Clinical isolate	Animal host-associated	GCF_000305215.1	10.1186/s13059-015-0701-6
**Proteobacteria**	Brevundimonas bacteroides DSM 4726	Obligate aerobe	Freshwater	Surface freshwater	GCF_000701445.1	10.1128/br.28.3.231-295.1964
**Proteobacteria**	Brevundimonas sp. AAP58	Obligate aerobe	Freshwater lake	Surface freshwater	GCF_001295975.1	SAMN02927139
**Proteobacteria**	Elstera litoralis	Unspecified aerobe	Enriched from lake diatom biofilm	Surface freshwater (phototroph-associated)	GCF_000963705.1	10.1099/ijs.0.026609-0
**Proteobacteria**	Nitrosomonas mobilis	Unspecified aerobe	Nitrifying granules	Built environment	GCF_900103035.1	10.3389/fmicb.2016.01869
**Proteobacteria**	Novosphingobium sp. AAP93	Obligate aerobe	Freshwater lake	Surface freshwater	GCF_001296055.1	SAMN02927144
**Proteobacteria**	Novosphingobium sp. NDB2Meth1	Obligate aerobe	Coal seam gas extraction bore well	Deep subsurface	GCF_900117425.1	SAMEA4535142
**Proteobacteria**	Porphyrobacter colymbi	Unspecified aerobe	Chlorinated swimming pool water	Built environment	GCF_002155685.1	10.2323/jgam.59.245
**Proteobacteria**	Porphyrobacter sp. CACIAM 03H1	Unspecified aerobe	Culture of Microcystis aeruginosa	Surface freshwater (phototroph-associated)	GCF_002215495.1	10.1128/genomeA.01069-17
**Proteobacteria**	Sandarakinorhabdus cyanobacteriorum	Unspecified aerobe	Aggregates of cyanobacteria in a lake	Surface freshwater (phototroph-associated)	GCF_002251755.1	10.1099/ijsem.0.002571
**Proteobacteria**	Sandarakinorhabdus sp. AAP62	Unspecified aerobe	Surface water of freshwater lake	Surface freshwater	GCF_000331225.1	10.1128/genomeA.00034-13
**Proteobacteria**	Erythrobacteraceae bacterium CCH12-C2	Unspecified aerobe	Showerhead biofilm	Built environment	GCF_001556995.1	10.1128/AEM.03529-15
**Proteobacteria**	Sphingomonas sp. CCH13-B11	Unspecified aerobe	Showerhead biofilm	Built environment	GCF_001557105.1	10.1128/AEM.03529-15
**Proteobacteria**	Sphingomonas sp. CCH15-F11	Unspecified aerobe	Showerhead biofilm	Built environment	GCF_001557005.1	10.1128/AEM.03529-15
**Proteobacteria**	Sphingomonas sp. CCH16-B10	Unspecified aerobe	Showerhead biofilm	Built environment	GCF_001556095.1	10.1128/AEM.03529-15
**Proteobacteria**	Sphingomonas sp. CCH18-H6	Unspecified aerobe	Showerhead biofilm	Built environment	GCF_001557405.1	10.1128/AEM.03529-15
**Proteobacteria**	Sphingomonas sp. CCH19-C6	Unspecified aerobe	Showerhead biofilm	Built environment	GCF_001556975.1	10.1128/AEM.03529-15
**Proteobacteria**	Sphingomonas sp. CCH20-B6	Unspecified aerobe	Showerhead biofilm	Built environment	GCF_001556565.1	10.1128/AEM.03529-15
**Proteobacteria**	Sphingomonas sp. CCH21-G11	Unspecified aerobe	Showerhead biofilm	Built environment	GCF_001555965.1	10.1128/AEM.03529-15
**Proteobacteria**	Sphingomonas sp. CCH5-A5	Unspecified aerobe	Showerhead biofilm	Built environment	GCF_001555785.1	10.1128/AEM.03529-15
**Proteobacteria**	Porphyrobacter donghaensis	Unspecified aerobe	Showerhead biofilm	Built environment	GCF_001556715.1	10.1128/AEM.03529-15
**Spirochaetes**	Leptospira ryugenii	Obligate aerobe	Surface water from a garden	Surface freshwater	GCF_003114855.1	10.1111/1348-0421.12671
